# Critical success factors for creating sustainable digital health applications: A systematic review of the German case

**DOI:** 10.1177/20552076241249604

**Published:** 2024-04-24

**Authors:** Lukas Schramm, Claus-Christian Carbon

**Affiliations:** Department of General Psychology and Methodology, 14310 University of Bamberg, Bamberg, Germany

**Keywords:** Digital health, DiGA, digital therapeutics, mhealth, ehealth, entrepreneur, leadership, dtx, digitalization

## Abstract

**Objective:**

The Covid-19 pandemic has accelerated the adoption of digital technologies to address social needs, leading to increased investments in digital healthcare applications. Germany implemented a special law called the “Digitales Versorgungsgesetz” (DVG—Digital Supply Act) in 2019, which enables the reimbursement of digital health applications, including digital therapeutics (DTx), through a fast-track process. The Federal Institute for Drugs and Medical Devices (BfArM), the German federal authority responsible for overseeing digital health applications, has implemented legislative adjustments since the law's introduction, which have increased requirements for these applications and potentially led to the removal of some from the directory as well as a slowdown in the addition of new ones. To counteract this trend, this work aimed to identify key success factors for digital health applications (DiGAs).

**Methods:**

This research identifies critical success factors through a structured literature review for developing sustainable digital health applications within the European healthcare systems, specifically DiGAs. The study aims to support the ongoing digital transformation in healthcare.

**Results:**

The identified success factors that significantly impact the sustainability of DiGAs include patient-centered design, application effectiveness, user-friendliness, and adherence to data protection and information security regulations using standardized approaches. These factors are crucial in preventing the failure of DiGA manufacturers in European countries.

**Conclusion:**

By considering and implementing these critical success factors, DiGA manufacturers can enhance their chances of long-term success and contribute to the digital transformation of the healthcare system in Europe.

## Introduction

The official announcement of the global Covid-19 pandemic on March 12, 2020^1^ and the accompanying policy measures have substantially changed social cohabitation and the economy. Thus, volatility, uncertainty, complexity and ambiguity of general conditions and situations accelerate even faster,^2^ which led to the circumstance that some disruptive technologies like telehealth or artificial intelligence (AI) developed from “nice to have” to “critical to have” or “must have” to address current and particularly upcoming challenges.^3^ This general trend is mainly observable in the healthcare industry.^4,5^ These new technologies and process changes came with social and industrial opportunities,^6^ of which were often used by entrepreneurs who were open to social innovations.^7^ This is particularly the case in the sector of digital health applications. Since the pandemic's start, it has shown a worldwide increase of 65%.^8^ Although some research exists on what innovations were developed as a rapid response, we still lack specific research on what success factors, business models, and entrepreneurial competencies led to those who survived the crisis and thrived in the healthcare market.^6,7,9,10^

Destruction is often accompanied by an excellent thrust for innovation, which must be used in an entrepreneurial way to operate successfully on the market. For this utilization, entrepreneurs must know their business's success factors. Until today, far less research has been done on what business models and social innovations will persist after the COVID-19 pandemic.^7^ Since Schumpeter already discovered that innovation is a driving force in the reconstruction and revival of growth, especially after a “gale of creative destruction”^11^ the present research aims to investigate success factors for DiGA manufacturers - the term used to describe the entrepreneurs and leaders who develop the applications and business models - use to create digital healthcare applications for the German healthcare industry. DiGA are medical software products certified under CE standards and undergo additional evaluation by the BfArM (Das Bundesinstitut für Arzneimittel und Medizinprodukte, i.e. Federal Institute for Drugs and Medical Devices) in Germany. Physicians and psychotherapists can prescribe DiGA to assist in the detection and treatment of diseases or facilitate the personalized implementation of treatment processes. These CE-certified digital medical devices belong to lower-risk classes. The costs associated with DiGA and any necessary medical services within their application are covered by statutory health insurance in Germany.^12^ Germany will be focused as it was the first country in the world to consistently integrate DiGA into the country's standard insurance healthcare^13^ and was followed by France and Belgium already. Therefore, this research specifically focuses on DiGA listed as such by the BfArM, as these are currently the only digital health apps that withstand German government-regulated verification and are reimbursed by health insurance funds. But will the recent developments become social innovations that displace old practices? What factors contribute to the limited number DiGAs reaching the reimbursement stage, and what are the critical success factors determining inclusion in the digital health applications’ directory and ensuring sustainable survival in the market for DiGAs? The five DiGAs with the most activation codes already account for 66 percent of all prescriptions across all 36 DiGAs listed in the reporting period of the Spitzenverband der Gesetzlichen Krankenversicherungen (GKV). These most frequently used DiGAs include Zanadio®, Vivira®, Kalmeda®, Somnio® and M-sense Migraine®. Although M-Sense® was removed from the DiGA list in April 2022, and the manufacturer of the DiGA Zanadio® Aidhere had to file for insolvency in May 2023. The Aidhere insolvency announcement came as a particular surprise as even in relative terms, i.e. taking into account the number of days in the DiGA directory, Zanadio tops the list with 40 activations per day and is already permanently listed.^14^ Web applications are also not included in this representation. Critical success factors may have an influence on the success of certain DiGAs.

### The present research approach

This review aims to identify the success factors of DiGA manufacturers. Therefore, we analyze relevant literature to design the missing competence framework for entrepreneurs in the field of DiGA. What differentiates the successful DiGA's from the other DiGA's that do not compete in downloads or prescriptions or did not even make it into the DiGA listing? At first, a structured literature review will be performed. This review will be based on the patient, intervention, comparison and outcome (PICO) schema as it is a common search strategy in the health sector for scientific literature research. The identified success factors are then verified with the help of artificial intelligence-based tools following the approach of Burger et al.^15^ and classified into technical, social, network management, organizational, ethical, and regulatory success factors. The derivation of success factor categories (technical, social, network management, organizational, ethical, and regulatory) in the context of DiGA responds to the developing nature of DiGA, lacking pre-established categorizations due to their recent emergence. Current research on DiGA primarily concentrates on establishing evidence, often overlooking categorizations for success factors. Notably, existing categorizations for startups and healthcare information systems are informative but risk being overly broad for the specific context of DiGA. The initial categorization aligns with a PESTEL analysis, adapting it to the DiGA domain to address this. The acronym PESTEL represents factors encompassing the political, economic, social, technological, ecological, and legal dimensions. In the methodological framework of our study, PESTEL analysis was chosen deliberately due to its ability to break down and interpret the factors systematically. With this approach, finding ways to position a company, or, in our case, a DiGA manufacturer, to survive in a dynamic business environment is possible.^16^

## Research design and methods

This research project aims to identify the success factors that enable entrepreneurs to exploit entrepreneurial opportunities in times of change and to develop a comprehensive framework that shows how DiGA can find its way to survive in the market. The research aims to pinpoint the entrepreneurial success factors that have become more relevant in Germany due to the changes in the regulatory framework given by the BfArM and evaluate their criticality for entrepreneurial success. Parameters such as the requirements for security, functionality, and quality, including interoperability, as well as the requirements for data protection and data security, are also taken into account, even if a large number of directives such as Social Insurance Code V, Medical Device Regulation, General Data Protection Regulation, and Digital health applications regulation specifies their implementation. To reach this goal, a structured literature review was performed, and primary and secondary sources were used. The research questions will be refined using a structured literature review following Tranfield, Denyer and Smart's^17^ approach but based the search string on the PICO schema. With this approach, we aim to minimize bias through exhaustive literature searches of published studies and to provide an audit trail for the reviewers’ decisions, procedures and conclusions.^18^ Previous research has focused on the medical patient benefits rather than the entrepreneurs behind DiGA, resulting in a lack of literature in this area. To fill this gap, we also include work from governmental agencies, experts from the field and reputable companies. In this way, “pragmatic science” strikes a balance between – in our case – the “clinical” rigor and “medical-economic” relevance and provides a timely topic through theoretical synthesis.^19^ This benefits scientists in the review process and entrepreneurs in developing a knowledge base, leading to added value for the academic and practitioner community.^17^ In addition, PICO searches show a greater number of hits in comparison to SPIDER searches.^20^

**Figure 1.**
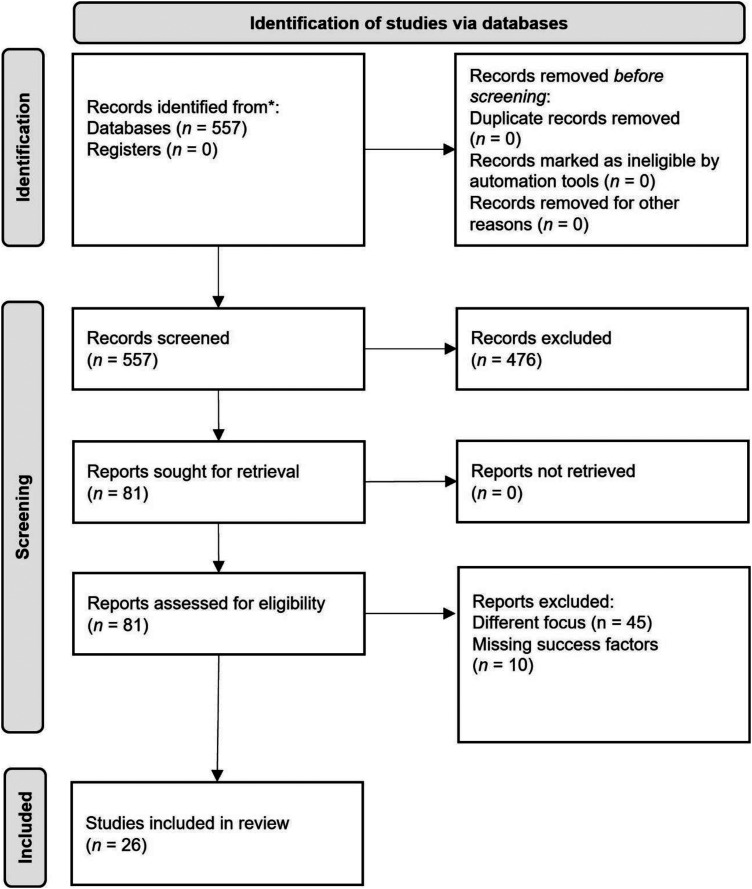
Preferred reporting items for systematic reviews and meta-analysis (PRISMA)^21^ flow chart for the study selection process on success factors for digital health applications.

After the search is completed, the search results are subjected to a post-processing phase and an analysis of the search data. This phase involves the identification of relevant papers, which are then subjected to two review loops for further analysis. Subsequently, the results are reviewed using artificial intelligence (AI), and a final synthesis of the findings is conducted. The review process is shown as a PRISMA flow chart in Figure 1.^21^

### Data collection procedures -PICO search

The search string is based on only three classes instead of four since the class “comparison” did not apply to this study, similar to the research of Granja et al.^22^ The other classes were defined as (P) healthcare manufacturer, (I) DTx and (O) success or failure. All terms of each class were combined with the logical OR operator and linked with the AND operator. This led to the following search query on February the 24^th^ 2023:

“TS = (Founder OR Entrepreneur OR start-up OR start up OR start ups OR startup OR leader OR business) AND TS = (Digital health application OR DIGA OR DTx OR Digital therapeutics OR mhealth OR ehealth OR digital intervention) AND TS = (success OR win OR fail OR lose OR failure OR useability OR lessons learned OR experience OR Barriers)”

In the context of a structured literature review, the inclusion of additional search terms next to DiGA, such as *ehealth*, *mhealth*, or *digital therapeutics,* in the search query is warranted to broaden the scope of research. This approach is justified by the limited existing scholarly publications on the success factors of DiGA, given their relatively recent emergence as a research domain. Incorporating these additional terms facilitates a more comprehensive identification of pertinent studies and helps address potential research gaps. Notably, these keywords were found in papers specifically related to DiGA, underscoring their relevance in the research context.

### Search data analysis

**Figure 2.**
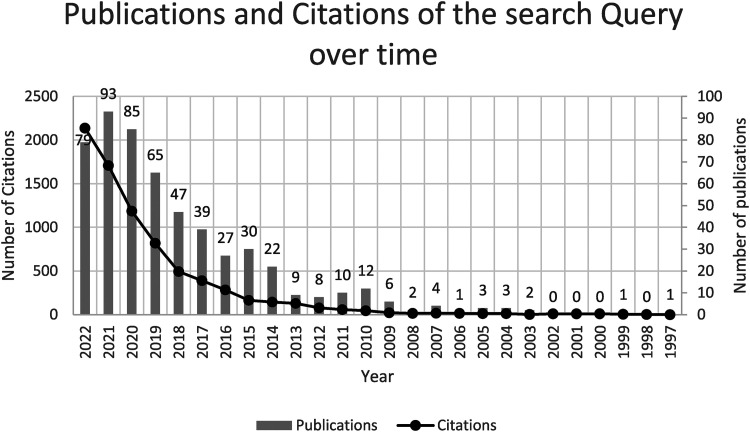
Number of publications and citations over the last three decades for the corresponding search string (own representation based on Web of Science Core Collection database search results; effective date February 24, 2023).

We used Web of Science as our database, as it is an independent database that ensures high-quality articles. The topic's novelty means it does not need to be limited in time by corresponding filter settings in the search, delivering 557 hits on the date mentioned. The fact that the search is not temporary nor locally limited also shows the increasing scientific interest in the topic, especially in the year of the introduction of the DVG in 2019 and the start of the Covid-19 pandemic, as Figure 2 shows.

**Figure 3.**
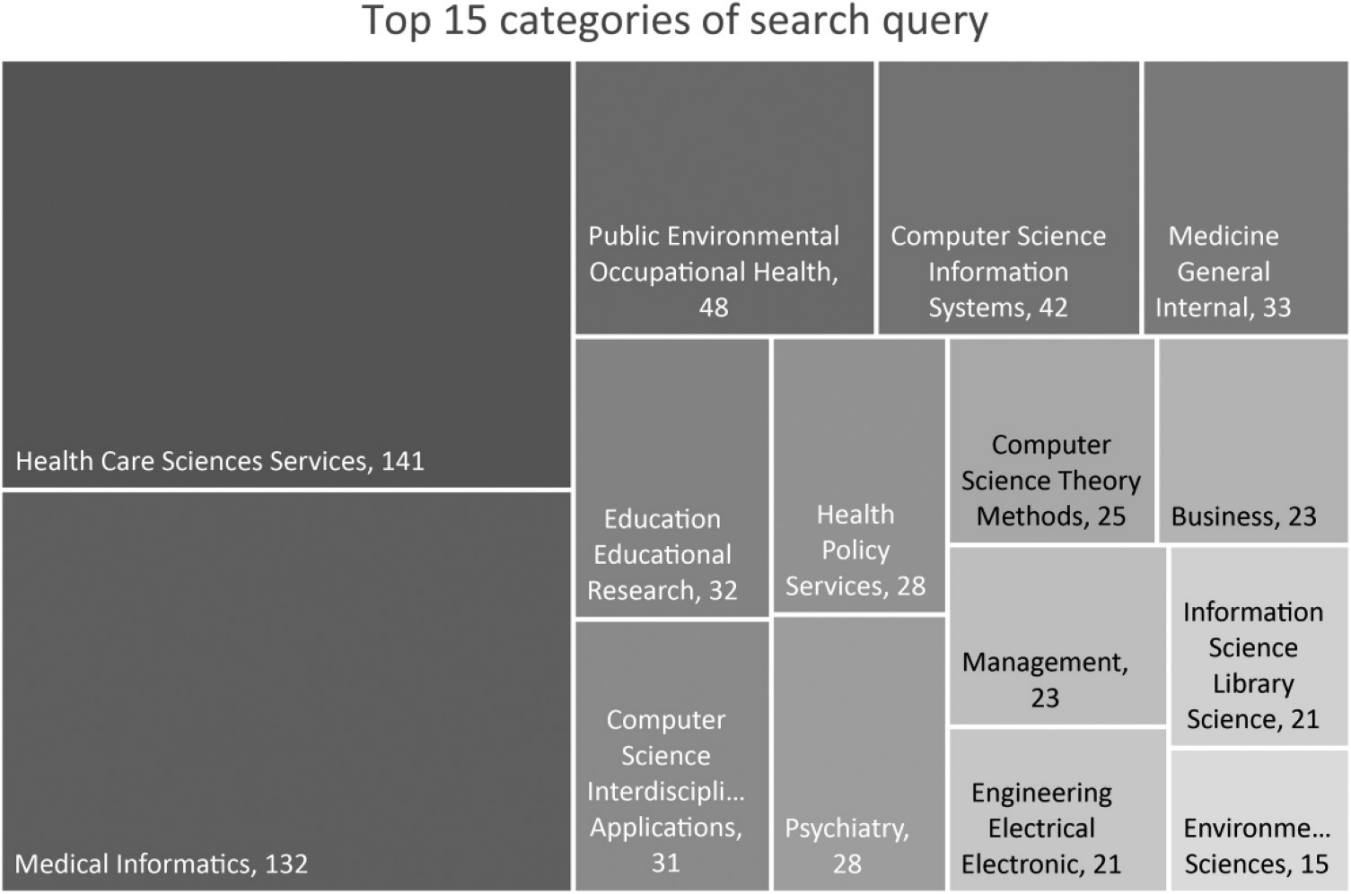
Top 15 web of science categories for the corresponding search string (own representation based on search results from Web of Science Core Collection; effective date February 24, 2023).

When filtering the results by categories, we found no business or management-related category among the top 10 categories. First, if we expand the list to include the top 15 categories, some business-related topics appear, as shown in Figure 3. The small number of scientific articles in management or business-related fields indicates the necessity of this topic and the current gap that researchers and entrepreneurs must tackle.

Further analyses of the business and management categories underline the growing demand for qualitative and quantitative research through increasing publications and citations.

### Data review 1 & 2 and analysis 1

After the general analysis of the search results, the individual articles were examined for their relevance to the research question. The first step was to screen the abstracts. In the screening, 81 papers qualified for closer selection. After a second round in which less relevant papers were sorted out, 36 papers remained. After the analysis, success factors from 26 papers were considered, as shown in Figure 1. The focus of the analysis of the papers was on the success factors that are crucial for a sustainable DiGA. Due to the limited literature, papers that were not restricted to digital therapies were also considered. The analysis period ran from the 24^th^ of February to the 2^nd^ of April 2023.

### Prompt generation

The identified success factors were then reviewed until 6^th^ of June using the generative AI of ChatPDF and additional factors added by the AI were re-evaluated and added to the results in order to ensure a high analysis quality. In addition, the risk of bias in the included studies could be counteracted in this way. To enable structured processing of the AI results, the following prompt was iteratively developed and tested against the results of ChatGPT before it was used in ChatPDF. The background to this approach is the practical possibility of analyzing entire PDF documents in ChatPDF, whereas in ChatGPT text had to be copied from the original. Both AI's ChatPDF and ChatGPT were based on a GPT-3.5 architecture. At the onset of this analysis, ChatGPT 4.0 was not yet available, thus to maintain consistency, the work was conducted using ChatGPT 3.5 instead. It became apparent that a two-step procedure is preferable for the analysis. In the first step, the understanding of the paper, author, methods and success factors was queried, and the role of the AI as a digitally assisting scientist was determined. The following prompt was used for this:

“What are the success factors in “[Title of the paper]” for digital health applications. Consider the authors’ research question, methodology, and findings, and evaluate the relevance and significance of the identified success factors. Based on the analysis, summarize the key insights and implications for the development and implementation of digital health applications”

In the second step, the following prompt is used to structure and systematically process the results, which significantly simplifies further handling of the results.

“List the success factors as bullet points for the technical factors, social factors, network management, organizational factors, ethical factors and regulatory factors”

### Analysis 2

The second prompt already provides dimensions for mapping, which do not restrict the results after checking. However, a human check of the results is necessary since the ChatPDF sometimes assigns the factors to different dimensions. This is the case, for example, if there are no indications of the dimension in the context or if a factor can apply to several dimensions. This procedure was applied to all 26 papers and led to uniform results. In some cases, ChatPDF could not identify success factors because they were not explicitly named in the paper. Nevertheless, critical success factors could be identified with follow-up questions resulting from ChatPDF's answer in these cases. To validate referencing the identified factors with their corresponding text passages in the PDF document a third prompt was used.

“In the analysis of the PDF file, precision is crucial. Please provide only the success factors present in the examined PDF and quote the exact text that led to the identification of the factor to facilitate accurate referencing. Avoid introducing fictional information and ensure that each statement is based on concrete content within the PDF. The utmost importance is placed on the accuracy and reliability of the information provided.”

**Figure 4.**
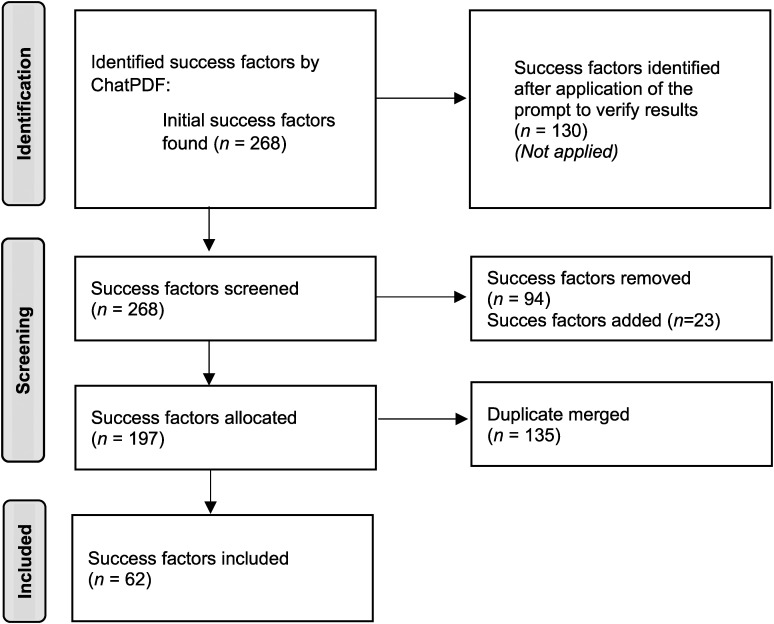
Illustration of the process of knowledge synthesis to identify success factors (own representation).

ChatPDF initially identified 268 success factors in the initial search, which was reduced to 130 after applying the third prompt. In cases where a referenced text passage did not align with the identified success factor, we checked if the factor could be found elsewhere in the text. In some instances, the factor was indeed present in the analyzed literature, raising the question of why ChatPDF cited a fictional passage. When the query did not function correctly, or ChatPDF issued an error message, the query was adjusted accordingly. In all cases, all 268 factors identified by ChatPDF were manually reviewed, excluding 94 fictitious success factors. Conversely, 23 factors not explicitly mentioned by ChatPDF were manually added. The process is illustrated in Figure 4.

The next step was to synthesize the identified factors and mapping them to the dimensions already mentioned in the prompt. The categorization of success factors into distinct dimensions (technical, social, network, organizational, ethical, and regulatory success factors) allows for a comprehensive understanding of the multifaceted aspects influencing the success of digital health applications (DiGAs). The advantages of this segmentation lie in its ability to systematically organize and analyze factors, providing clarity and aiding in targeted interventions within specific dimensions. At this point, the remaining 197 success factors were categorized, and instances of multiple mentions of a factor were consolidated. After this step, 62 success factors remained, as depicted in Figure 4.

The knowledge synthesis process involved leveraging suggestions from ChatPDF for mapping success factors to the designated dimensions. While a substantial portion of the suggested mappings by ChatPDF were adopted, manual adjustments were necessary to refine the categorization accurately. This iterative approach ensures a nuanced and reliable representation of the success factors within their respective dimensions, as Table 1 shows. In our study, the initial categorization of success factors for digital health applications was derived from the PESTEL Framework, and subsequently refined and validated using ChatGPT, to ensure relevance and comprehensiveness. This dual approach, integrating traditional analysis with advanced computational tools, aimed to enhance our findings’ methodological rigor and applicability. The exclusion of political factors is justified by the comprehensive regulatory framework established by the BfArM. Economic factors are consolidated into organizational considerations, environmental factors are redefined as network factors, and ethical considerations are explored as an independent dimension. Social, technical, legal, or regulatory factors remain unchanged but do not focus on macro but on micro factors. These delineated success factor categories function as a fundamental framework specifically tailored to the distinctive challenges and dynamics characterizing the DiGA landscape. The need for further research to validate arises from the manual interventions and is crucial to affirm the accuracy and reliability of the results.

**Table 1.** Results of the structured literature review: critical success factors in the development, deployment, and distribution of digital health applications.

Technical Factor	Social Factor	Network Management	Organizational Factor	Ethical Factor	Regulatory factor
Design and functionality that is user-friendly/centered and meets the (individual) needs of patients and healthcare professionals^1^^,^^3^^,^^5^^,^^6^^,^^9^^,^^11^^,^^12^^,^^18^^,^^19^^,^^20^^,^^23^^,^^24^^,^^25^^,^^26^	Involving end-users in the design process^3^^,^^6^^,^^9^^,^^11^^,^^12^^,^^17^^,^^20^	Ensuring all stakeholders are involved in the development and implementation process^1^^,^^4^^,^^5^^,^^6^^,^^9^^,^^10^^,^^11^^,^^17^^,^^20^	Structures and processes within healthcare organizations that facilitate adoption, such as leadership support, resource allocation, and workflow integration^1^^,^^11^^,^^21^^,^^23^^,^^20^^,^^25^	Consider privacy and confidentiality and obtain informed consent from end-users, e.g. for data collection and use (e.g. by maintaining high standards of privacy, security and effectiveness).^3^^,^^6^^,^^7^^,^^8^^,^^19^^,^^13^^,^^21^^,^^25^^,^^26^^,^^1^^,^^5^^,^^9^^,^^16^^,^^23^	Complying with relevant laws, regulations, and standards for data collection, storage, and use^2^^,^^3^^,^^4^^,^^5^^,^^6^^,^^7^^,^^13^^,^^14^^,^^19^^,^^21^^,^^25^
Address privacy and security concerns to build user trust and ensure data security and privacy, e.g. through appropriate encryption, access controls and data sharing agreements^1^^,^^6^^,^^26^^,^^2^^,^^3^^,^^7^^,^^8^^,^^9^^,^^12^^,^^13^^,^^14^^,^^21^^,^^25^	Taking into account the different cultures and age groups as well as linguistic diversity among end users and developing a common language^4^^,^^11^^,^^12^^,^^19^^,^^21^^,^^23^	Establish strong networks and partnerships^2^^,^^3^^,^^4^^,^^5^^,^^9^^,^^13^	Offering ongoing training, education, and support to help doctors^14^^,^^3^^,^^19^^,^^20^^,^^23^^,^^25^	Ensuring ethical use of patient data and respect for patient autonomy^2^^,^^4^^,^^7^^,^^8^^,^^19^	Be aware of relevant regulations and work closely with legal experts or regulatory bodies^2^^,^^4^^,^^5^^,^^7^^,^^12^
Usability and interoperability to ensure effective use by all stakeholders (e.g. EHR electronic health record)^1^^,^^4^^,^^5^^,^^6^^,^^7^^,^^20^^,^^21^^,^^24^^,^^25^	Education, training and on-boarding of end users^23^^,^^24^^,^^25^^,^^26^^,^^22^^,^^3^	Effective communication and coordination among stakeholders^4^^,^^8^^,^^10^^,^^11^^,^^12^^,^^25^	Integration into existing workflows, policies and organizational goals to ensure seamless adoption and use^1^^,^^3^^,^^20^^,^^21^	Addressing issues related to data ownership, stewardship, and disposal^3^^,^^4^^,^^13^	Monitoring compliance with regulatory requirements throughout the app development process^3^
Providing technical support for end-users^3^^,^^19^^,^^20^^,^^23^^,^^26^	Prove clinical effectiveness and/or address societal or patient benefits^14^^,^^23^^,^^25^^,^^26^	Access to mentors and advisors^4^	Developing a sustainable revenue model^5^^,^^9^^,^^13^	Consider ethical implications when designing digital health applications, such as potential biases or unintended consequences^12^	
Scalability^4^^,^^5^^,^^7^^,^^10^^,^^21^	Building trust with patients and healthcare providers^2^^,^^4^		Clear governance structures and decision-making processes^6^^,^^7^^,^^8^	Maintaining transparency in business practices and data collection^2^	
Use of agile development methodologies (e.g. iterative development)^1^^,^^6^^,^^10^^,^^11^	Understanding patient needs and preferences, including patient engagement, provider buy-in, and cultural norms around healthcare^1^^,^^2^		Funding and resource allocation^10^^,^^11^^,^^22^		
Resolve bandwidth, load time, latency and data retention issues and ensure reliable connectivity, data transfer and uptime^3^^,^^7^^,^^21^	Patient engagement and empowerment^7^^,^^24^		Provide training or resources to help team member^17^^,^^18^		
Conducting thorough testing to ensure functionality and usability^3^^,^^11^	Improved patient-provider communication and collaboration^7^		Alignment of digital health services with existing care processes^8^^,^^24^		
Use robust standards and technology infrastructure development^7^^,^^16^	Encourage team members to voice their expectations at the start of the project to prevent miscommunication at a later stage^12^		Strong leadership^4^^,^^6^		
Ease of use^16^^,^^20^	Developing services that align with doctors’ attitudes towards technology and innovation^14^		clear vision and mission statement^4^^,^^10^		
Clear value proposition^13^	Providing opportunities for doctors to try out new technologies^14^		Flexibility and adaptability^4^^,^^18^		
Provide more feedback to app developers^15^	Offering solutions that help doctors establish their personal brand^14^		Adopting a flexible approach to business model design^5^^,^^10^		
Incorporate functionalities that support patient-provider relationships^15^	Connect users with others who share similar health concerns or goals^16^		focus on doctors who have a positive attitude towards mHealth services and are highly innovative^14^		
Ensuring software compatibility and hardware specifications^3^	Developing a supportive culture of innovation and continuous improvement^25^		Ongoing monitoring, evaluation and adaptation of systems^17^		
			Workplace readiness: stakeholder acceptance and engagement^22^		
			Access to internal expertise^25^		
			Effective marketing strategies^13^		
			Effective customer relationship management^13^		
			Understanding the general dynamics of currently existing commercialization attempts and business models^9^		
			Prioritization of longitudinal health records and digital interactions with patients^7^		
			Establishing clear roles and responsibilities for all stakeholders^5^		
			Developing effective management strategies^2^		

^1^ Swinkels et al.,^23^; ^2^ Muhos et al.,^24^; ^3^ Fillo et al.,^25^; ^4^ Lim and Anderson,^26^; ^5^ Oderanti et al.,^27^; ^6^ Dalton,^28^; ^7^ Neinstein et al.,^29^; ^8^ Vannieuwenborg, Verbrugge and Colle,^30^; ^9^ Oderanti and Li,^31^; ^10^ van Limburg et al.,^32^; ^11^ Bhattacharyya et al.,^33^; ^12^ Vermeulen et al.,^34^; ^13^ Vimarlund, Nikula and Nøhr,^35^; ^14^ Hu, Fang and Wang,^36^; ^15^ Lan et al.,^37^; ^16^ Li et al.,^38^; ^17^ Ross et al.,^39^; ^18^ Simon et al.,^40^; ^19^ Carlqvist et al.,^41^; ^20^ Kujala et al.,^42^; ^21^ Sezgin, Özkan-Yildirim and Yildirim,^43^; ^22^ Alami et al.,^44^; ^23^ Semple et al.,^45^; ^24^ Bauer et al.,^46^; ^25^ Polhemus et al.,^47^; ^26^ Peek et al.^48^

The entire process for identifying and selecting success factors is illustrated in Figure 4.

## Results

The results of the structured literature review are shown in Table 1. The results are sorted in descending order of relevance. The relevance of a factor depends on the number of references and should be verified in further research. The numbering in the table refers to the table and not to the body text.

**Figure 5.**
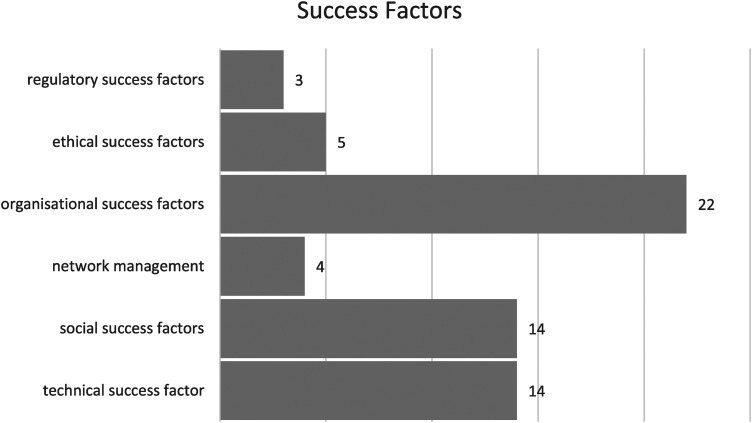
Technical, social, network management, organizational, ethical, and regulatory success factors.

Overall, 62 success factors have been identified. These 62 factors consist of 14 technical, 14 social, 22 organizational, four factors of network management, five ethics, and three regulation (Figure 5).

### Technical success factors

What is striking about the technical factors is that more than half of the identified papers stated that patient-centered or user-friendly design is a key success factor in DiGA development. Ensuring functionality and usability and providing feedback to the developers is essential to ensure this factor. The second technical factor on data protection and information security is also very striking, as this topic is generally mentioned the most in the ethical and regulatory factors. The issue of interoperability and ease of use, which allow a variety of actors to interact with the application, e.g. when it comes to electronic health records, is also mentioned several times. The availability of a technical support that can interact with patients, doctors and other stakeholders is also supported by the social factor, which indicates the interaction and communication between user and provider.

### Social success factors

The social factor, which is mentioned most often, can be seen as an enabler for the technical factor on user centered design. It states that patients should be involved in the design process and that cultural, linguistic and age differences between end-users should be taken into account to get a user-centric perspective. The user-centered approach is also supported by education, training and onboarding for end users, building trust with patients, understanding patient needs and preferences, and patient engagement and empowerment. The factor of proving clinical effectiveness and/or address societal or patient benefits is consistent with the medical benefit and or patient-relevant structure and procedure improvements required by the BfArM for DiGA.

### Network management

Similar to the involvement of end users in the design process, including all stakeholders in the development process becomes visible as a success factor. This stakeholder involvement also speaks for the second and third identified factor of creating strong networks and partnerships and effective communication and coordination among the stakeholders. Further factors mentioned here are access to mentors and advisors.

### Organizational success factors

Organizational factors include structures and processes within health organizations that facilitate adoption, such as leadership support, allocation of resources and integration of workflows, goals and policies. As 89% of DiGA prescriptions are prescribed by doctors,^14^ who are most likely to come into contact with patients, it also makes sense to offer training, education and support to doctors. The factor of focusing on doctors with a positive attitude towards mhealth also refers to the doctor-provider relationship. In the further section on organizational factors, a number of rather general factors are mentioned, which are often not supported by a second source. These will not be discussed further here.

### Ethical success factors

In terms of ethical and regulatory factors, cybersecurity and privacy issues are certainly at the forefront of gaining the trust of patients and physicians, e.g. by providing informed consent and applying standards for data collection, storage, and processing. The topic of transparency in business practices and data management is also mentioned among ethical factors.

### Regulatory success factors

Regulatory factors focus on data protection, information security and compliance with regulatory data collection, storage, and use standards. Close cooperation with legal experts and regulatory authorities such as the BfArM is crucial to ensure legal adherence throughout the development and roll-out process.

## Discussion

The development, deployment, and distribution of regulated digital health applications poses many challenges for entrepreneurs due to the number of regulations and the fast-moving and complex nature of the field. This structured literature review identified numerous technical, social, network management, organizational, ethical, and regulatory success factors using generative artificial intelligence based on the GPT 3.5 architecture. In order to select the suitable literature in advance, the PICO method was used to search for relevant literature. This literature was then narrowed down to 26 relevant papers in two review loops.

According to Faix,^49^ a high degree of entrepreneurship and leadership is needed today and in the future to stop the challenges of a fundamental nature through innovation in society and the economy. The 62 success factors identified in this paper are intended to help innovative entrepreneurs who face these new challenges to run their businesses sustainably and meet the social need for health. In order to achieve this, the results table provides entrepreneurs with a framework for orientation in the development, deployment and distribution of DiGA that encompasses several dimensions.

Regarding technological key success factors, there have already been great efforts in the creation of a mHealth app trustworthiness Checklist (mHAT) that can be modified for DiGA.^50^ For the modification, regulatory standards from the DiGA guideline could be added to the list to be more precise. Recent research published after the analysis period also confirms that usability is a key success factor of DiGA, whereas the importance of privacy issues for the end-user is relativized.^51^ Because regulations partly prescribe these data protection topics and non-compliance can result in expensive sanctions for the company, compliance is indispensable in most cases.

There are some limitations to this study, which we want to discuss in the following. The main weakness in this approach is the lack of verification of the results specifically for DiGA, e.g. through expert interviews. Due to a shortage of literature, the study is not based on articles on the topic of DiGA, but also examines less regulated digital health applications. In this study, the *Web of Science* databases were exclusively utilized for the structured literature review. This constraint may result in an incomplete coverage of specific subject areas and potentially contribute to selection bias. It is noteworthy that other pertinent sources may not have been considered, and, consequently, additional insights could be gleaned from alternative databases or information reservoirs. The categorization of success factors into different dimensions introduces certain limitations to this study. While this approach allows for a structured analysis, it may oversimplify the intricate interdependencies among success factors. Some factors may exhibit multidimensional characteristics, and the rigid classification might not fully capture their nuanced effects. These offer possibilities for further research. Furthermore, the results do not include papers published after the end of the analysis period. However, the topics considered relevant by the author team from a recent article by Uncovska et al.^51^ should be briefly mentioned here in the discussion, as they explicitly relate to regulated *mhealth* applications:

- Quickly resolve issues with basic functionality and the registration process.
- Active collection of user feedback and analysis and use of patient-generated data
- Communicate the effectiveness of the therapy, through practitioners, general information campaigns, regulators, health insurers as well as providers.
- Ensure that user experience is an integral part of the mHealth app review process.
- Improve patients’ understanding of the effectiveness of therapies through information campaigns for patients and practitioners.
- Ensure usability, personalization and customer service.

The findings obtained here confirm the results of the SLR.

Even in comparison to non-scientific literature, such as white papers and blog posts by German DiGA experts, there are some overlaps. Since the content here is explicitly focused on DiGAs, they should be considered in the discussion as potential success factors for DiGA manufacturers. Specifically, regarding regulatory success factors such as consulting services and collaboration with the right experts, the recommendations align with those found in white papers and research findings. Additionally, the focus on Risk Class 1 in manufacturing is emphasized. The success factors related to the distribution of DiGAs through offerings and a focus on physicians also align with the suggestions found in German white papers and blog posts, and they are further complemented by the inclusion of building a field sales force or collaborating with pharmaceutical companies to achieve a more targeted healthcare professional (HCP) access.^52,53^

Especially for existing pharmaceutical companies aspiring to venture into the realm of Digital Health Applications (DiGAs), additional success factors have been delineated in the literature concerning the integration of digital health within the pharmaceutical industry. These encompass the ability to respond swiftly to changes, adopt new, more flexible strategies, enhanced collaboration with long-standing and novel stakeholders, and increased transparency towards patients and regulatory bodies.^54^

In examining the categorization of success factors within the dimensions of the PESTEL framework, this study also endeavors to juxtapose and discuss established principles, such as those related to open innovation platforms. Notably, a subset of the identified success factors aligns well with principles such as collaborative onboarding, enforcing responsibilities, demonstrating appreciation, ensuring relevance, and facilitating mutual evolution. This alignment is particularly evident as some factors are explicitly mentioned within the definitions of these principles. However, the principles exhibit limitations in accommodating additional technical, ethical, and legal factors, indicating a gap in the categorization schema. While integrating these principles into the organizational, social, and network factors provides enhanced granularity and is deemed valuable for further research, it underscores the need for a more encompassing framework that can systematically incorporate a broader spectrum of success factors. This observation opens avenues for future research to refine and expand upon the categorization methods, potentially incorporating or adapting principles from open innovation platforms to enrich the understanding and structuring of success factors in digital health applications and beyond.^55^

## Conclusion

The 62 success factors identified in the analysis can be categorized into several key areas: technical, organizational, social, network management, ethical, and regulatory. These factors are crucial in DiGA development, deployment, and sales phases.

Technical factors encompass aspects such as designing user-friendly and functional applications that meet the needs of healthcare professionals and patients. Usability and interoperability, along with addressing technical issues like connectivity and data retention, are also important considerations.

Organizational factors involve structures and processes within healthcare organizations that facilitate adoption, including leadership support, resource allocation, and integration into existing workflows and policies. Developing a sustainable revenue model is also essential.

Social factors emphasize end-users’ involvement in the design process, education and training for patients and physicians, effective communication and coordination among stakeholders, and patient engagement and empowerment. Considering different cultures, age groups, and linguistic diversity among end-users is also an important quality for widespread adoption.

Network management factors highlight establishing strong networks and partnerships, collaborating with academic partners, and integrating digital health services with existing care processes.

Ethical factors emphasize the importance of maintaining privacy and confidentiality, obtaining informed consent, and ensuring the ethical use of patient data. Addressing potential biases and unintended consequences in the design of digital health applications is also recommended.

Regulatory factors encompass compliance with relevant laws, regulations, and data collection, storage, and use standards. Obtaining necessary approvals from regulatory bodies and working closely with legal experts or regulatory bodies are crucial to ensure adherence to regulatory requirements throughout the development and deployment process.

These success factors provide a comprehensive framework for developing and implementing digital health applications like DiGAs. By considering and addressing these factors, stakeholders can enhance their digital health solutions’ effectiveness, acceptance, and impact in the healthcare ecosystem.

In terms of policy and considering the findings, we suggest that regulators consider making user experience an integral part of the DiGA review process, as it is critical to uptake and compliance. On the other hand, there are very strict regulators that make DiGA development difficult and costly for manufacturers, but are not important to the patient, as the latest literature shows.^51^ Here, the authors recommend that policymakers in Germany and other countries find a solution that puts the patient's health interests first.
